# Epidemiological and clinical characteristics of mpox cases: correspondence

**DOI:** 10.1590/S2237-96222023000100026

**Published:** 2023-04-03

**Authors:** Rujittika Mungmunpuntipantip, Viroj Wiwanitkit

**Affiliations:** 1 Private Academic Consultant, Bangkok, Thailand; 2 Joseph Ayobabalola University, Ikeji-Arakeji, Nigeria

Dear Editor,

We would like to share ideas on the publication *Epidemiological and clinical characteristics of monkeypox cases in Brazil in 2022: a cross-sectional study* ^1^ The epidemiological and clinical characteristics of mpox documented by Pascom et al., in Brazil, were comparable to those of other nations, such as México and Nigéria.^1^ ^,^ ^2^ ^,^ ^3^ An epidemiological characteristic that has been common is the highest occurrence of the disease among males, especially homosexual men.^2^ ^,^ ^3^

The clinical manifestation of mpox is a crucial clinical aspect. The presence of both additional symptoms and occasional asymptomatic or mildly symptomatic individuals, without fever and with occult skin lesions, may hinder diagnosis.^4^ According to recommendations from the World Health Organization (WHO) and the U.S. Centers for Disease Control and Prevention (CDC), laboratory confirmation of samples of suspected mpox cases should be performed using nucleic acid amplification tests - such as real-time or conventional polymerase chain reaction - but also taking into consideration clinical and epidemiological information.^5^

Thus, although laboratory testing is the cornerstone of the diagnosis of mpox, laboratory quality control remains a problem.^5^ Indeed, the article by Pascom et al. presents a broader context, but an important point of discussion is the laboratory issue. Given that the data in most reports, including the article under discussion, are usually based on laboratory-confirmed cases of mpox, it is necessary to recognize the role of accuracy of their laboratory diagnosis. Laboratory results are usually the basis for surveillance, however, it is essential that they include repeat testing because of the possibility of false-negative results. A solid quality management system is urgently needed for the clinical laboratory services involved in the diagnosis of mpox.^6^

In addition to laboratory diagnosis, professionals’ awareness is important for success in early identification of mpox cases. A surveillance system with reliable and updated data depends on the adequate diagnosis and reports made by the professionals involved. For its strengthening, it is necessary the training of all professionals who are responsible for the primary notification, as well as the certification about the quality of laboratories. A case reassessment system and internationally shared country reports can also contribute.^7^

In a scenario where the disease is new, the lessons from the country that has just reported the first case can be very useful. In Singapore, for example, the recommendation emerged from local experience is for robust surveillance mechanisms; continued partnership between public health and frontline professionals, reaching at-risk communities through existing networks, offering testing and reducing barriers to health care are crucial to outbreak control.^8^ This may also be applicable to Brazil and other South American countries.
